# Adult-Onset Still’s Disease After the ChAdOx1 nCoV-19 Vaccine

**DOI:** 10.7759/cureus.21279

**Published:** 2022-01-15

**Authors:** Elham A AlQudari, Lulwah I Alabdan, Abdulla A Alkhathami, Mohammed D Alotaibi, Hanan A Alhamzi

**Affiliations:** 1 Department of Medicine, Prince Mohammed bin Abdulaziz Hospital, Riyadh, SAU

**Keywords:** autoimmune disease, vaccine, covid 19 side effect, covid 19, adult-onset still’s disease

## Abstract

Adult-onset Still's disease (AOSD) is a rare autoimmune disorder without clear etiology. It is known that genetic and infectious causes trigger diseases. AOSD cases have been reported after coronavirus disease 2019 (COVID-19) infection and post influenza vaccine.

Here, we report this challenging case of adult-onset Still's disease in a patient who recently received the ChAdOx1 nCoV-19 vaccine (Oxford-AstraZeneca).

## Introduction

Adult-onset Still's disease (AOSD) is a systemic inflammatory disorder of unknown etiology. Patients present with fever, arthritis, and evanescent rash. Sore throat, hepatomegaly, splenomegaly, lymphadenopathy, and serositis are other frequent clinical features [1]. Both genetic factors and infectious triggers have been suggested. Still, there has been no evidence of an infectious etiology, and data for the genetic factors fluctuate [2]. AOSD is relatively benign, but life-threatening complications have been described, including macrophage activation syndrome (MAS), disseminated intravascular coagulation (DIC), and thrombotic thrombocytopenic purpura (TTP) [3].

However, coronavirus disease 2019 (COVID-19) is caused by the severe acute respiratory syndrome coronavirus 2 (SARS-CoV-2) virus. COVID-19 has several clinical and laboratory features similar to AOSD, including systemic inflammation, fever, high serum ferritin, and a potentially life-threatening cytokine release syndrome [4]. The first case of AOSD and COVID-19 was reported by de Carvalho [5]. The diagnosis was challenging, but the outcome was favorable.

In this report, we present a challenging case of AOSD in a patient who had recently received a COVID-19 vaccine.

## Case presentation

We report the case of a 29-year-old Indian man without any comorbidities or family history of autoimmune diseases. He developed a fever, headache, sore throat, myalgia, and arthralgia two days after receiving the first dose of the COVID-19 vaccine (Oxford-AstraZeneca). He sought medical advice and was treated with antibiotics and an antipyretic for five days without improvement. He presented to the emergency room of Prince Mohammed Bin Abdulaziz Hospital in Riyadh, Saudi Arabia. Upon admission, he was febrile with tachycardia; his pulse was 130 beats/min, his oral temperature was 38 °C, his blood pressure was 119/71 mmHg, and oxygen saturation was 94% on room air. Other elements of his physical examination were unremarkable. His serum inflammatory markers were elevated, as shown in Table 1.

**Table 1:** Laboratory results

Laboratory test	Result	Normal range
White Blood Count	26.2 x10^9 / L	4.00 -11.00 x10^9 / L
Neutrophil	22.90 x10^9 /L	1.5-7.8 x10^9 per L
Lymphocyte	1.35 x10^9 / L	0.85-4.10 x10^9 / L
Monocyte	1.29 x10^9 /L	0.2-1.10 x10^9 / L
Hemoglobin	110 g/L	135 - 175 g/L
Erythrocyte Sedimentation Rate	120 mm/h	0 - 15 mm/h
C-reactive Protein	>16 mL/dl	0.01 - 0.50 mL/dl
Ferritin	>2000 ng/ml	21.8 - 274.6 ng/ml
D-dimer	12 ug/mL	0.00 - 0.50 ug/mL
Aspartate Transaminase	70 units/L	5-34 units/L

His septic workup, which included blood and urine culture and urinalysis, was negative, and he tested negative three times for SARS-CoV polymerase chain reaction. An empirical antibiotic (ceftriaxone and azithromycin) was started, but the patient's fever persisted. Ten days later, non-pruritic skin rashes began to appear on his face, trunk, and extremities, with bilateral knee pain and swelling. His fever reached 40 °C, and he became hypotensive and required oxygen with bilateral chest crepitation. Repeated septic workups showed no evidence of infection. Antinuclear antibodies (ANA) and rheumatoid factor (RF) were also negative, and his echocardiogram was normal. A peripheral blood film showed no evidence of leukemia. Chest, abdomen, and pelvic computed tomography (CT) with contrast were performed and showed extensive bilateral perihilar airspace consolidation and ground-glass opacities; however, there was no evidence of pulmonary embolism, malignancy, hidden collection, significant lymph node enlargement, or splenomegaly.

After a discussion among the rheumatology, infectious disease, and hematology teams, and since there was no evidence of ongoing infection or malignancy and the patient fulfilled the Yamaguchi criteria (with 4/4 major criteria and 3/4 minor criteria, based on the exclusion criteria), a diagnosis of AOSD was made, and he was started on 1 mg/kg methylprednisone daily. Although his fever subsided within two days, his oxygen saturation decreased to 91% with more lung infiltrates. Later, the patient developed cardiovascular arrest. He was revived and connected to mechanical ventilation. The dose of methylprednisolone was increased to 1000 mg daily for three days, followed by 100 mg daily. The patient showed improvement within three days and was extubated by Day 4. One day later, his oxygen saturation was 99% on room air. The patient's skin rashes disappeared, and his joint pain resolved. His C-reactive protein levels returned to normal, and his liver function test normalized. He was discharged on tapered doses of oral prednisolone and, at one-month follow-up, he was doing well.

## Discussion

AOSD is a well-known, though rare, systemic inflammatory disease. At present, its etiology is unknown, though there is evidence that environmental factors with genetic predisposition play a role [1]. The association between AOSD and infectious agents has been discussed on several occasions [6]. Several viral infections, such as the Epstein-Barr virus, cytomegalovirus, human herpesvirus 6, influenza virus, parainfluenza viruses, and Coxsackie virus, have been reported as possible triggers [7]. AOSD cases have been reported after COVID-19 infection and influenza vaccinations [4,8]. In July 2021, the first case of AOSD was written about following a COVID-19 vaccine. In that case, a causal relationship was suspected on the basis of the solid temporal association between the vaccine and the patient's symptoms [9]. An association between vaccines and autoimmune diseases is a well-described phenomenon based on a temporal relationship. The vaccination should precede the earliest manifestation of the event to establish a solid relationship [8]. In the case that we reported, there was a strong temporal association and an absence of alternative causes, leading us to suspect a causal relationship between the COVID-19 vaccine and AOSD.

Our case highlights the possibility of autoimmune activation after COVID-19 vaccination. The risk of post-vaccination autoimmune disease has been discussed in many studies. However, strong associations with particular vaccines have been rare such as acute disseminated encephalomyelitis following a rabies vaccine, cases of Guillain-Barre neuritis following a swine influenza vaccine, and autoimmune thrombocytopenia described after a measles vaccination [10]. In our case, the patient developed characteristic symptoms of AOSD with pneumonitis a few days after receiving a COVID-19 vaccine.

The mechanism by which a vaccine can trigger an autoimmune/autoinflammatory disease may be related to the similarity between microbial (vaccine) molecules and a patient's self-antigens (antigenic mimicry) or by vaccine molecule-related signals that trigger innate immunity and overcome the regulatory mechanisms that prevent autoimmune diseases [8].

AOSD shares not only clinical features but also common pathogenic pathways with COVID-19. The typical role of Interleukin 1 (IL-1 ) in the pathogenesis of AOSD and COVID-19 could explain the close similarities between the diseases, and it has been postulated that a potentially misdirected immune response against SARS-CoV-2 can trigger the onset of AOSD in some cases [4].

## Conclusions

AOSD is a rare condition, but it can occur after vaccination. Therefore, it is necessary to consider working up autoimmune diseases with the appearance of fever and a rash after vaccination without an apparent source. Furthermore, early diagnosis and treatment improve the outcome and prevent complications.
